# Digital Twinning of Hydroponic Grow Beds in Intelligent Aquaponic Systems

**DOI:** 10.3390/s22197393

**Published:** 2022-09-28

**Authors:** Abraham Reyes Yanes, Rabiya Abbasi, Pablo Martinez, Rafiq Ahmad

**Affiliations:** 1 Aquaponics 4.0 Learning Factory (AllFactory), Department of Mechanical Engineering, University of Alberta, Edmonton, AB T6G 2G8, Canada; 2 Department of Mechanical and Construction Engineering, Northumbria University, Newcastle upon Tyne NE1 8ST, UK

**Keywords:** digital twin, IoT, precision farming, aquaponics farm 4.0

## Abstract

The use of automation, Internet-of-Things (IoT), and smart technologies is being rapidly introduced into the development of agriculture. Technologies such as sensing, remote monitoring, and predictive tools have been used with the purpose of enhancing agriculture processes, aquaponics among them, and improving the quality of the products. Digital twinning enables the testing and implementing of improvements in the physical component through the implementation of computational tools in a ‘twin’ virtual environment. This paper presents a framework for the development of a digital twin for an aquaponic system. This framework is validated by developing a digital twin for the grow beds of an aquaponics system for real-time monitoring parameters, namely pH, electroconductivity, water temperature, relative humidity, air temperature, and light intensity, and supports the use of artificial intelligent techniques to, for example, predict the growth rate and fresh weight of the growing crops. The digital twin presented is based on IoT technology, databases, a centralized control of the system, and a virtual interface that allows users to have feedback control of the system while visualizing the state of the aquaponic system in real time.

## 1. Introduction

Aquaponics is a farming method that combines the use of recirculating aquaculture systems (RAS) and hydroponics, growing symbiotically aquatic organisms and plants [1]. As an overview, the waste of aquatic animals in the aquaculture tanks contains dangerous levels of ammonia, if allowed to accumulate. In aquaponic processes, the ammonia-enriched effluent is taken towards biofilters where nitrifying bacteria break down ammonia into nitrites and nitrates. The nitrates then produced can be used as plant nutrients. After that, all the water is recirculated again to the aquaculture tanks safely [2]. The symbiotic process of aquaponics makes this technology green, sustainable, and highly desirable to tackle food security. Therefore, it is getting increased attention from researchers and practitioners alike because of its ability to save resources while offering high efficiency [3].

Although aquaponics shows an immense potential, it has become difficult for producers to adopt this system due to the economic feasibility at the commercial scale [4]. For example, it is determined that RAS is two to three times more expensive than raising fish in open ponds [5]. Another main consideration that limits aquaponic systems of being profitable is the current required workforce. As these systems are quite complex due to their multiple biological components and requirements, such as disease prevention or monitoring water quality and levels, the workload is considerable, leading to continuous rigorous inspections seven days a week, 24 h per day [5]. Estimations made by Tokunaga et al. state that, in aquaponic environments, labor costs can reach around 46% of the total operating costs and about 40% of the total annual costs [6].

Under this scenario, it seems feasible to start working towards the automation, control, and implementation of smart systems in aquaponics to reduce labor costs and increase profitability by adopting precision agriculture [7]. Recently, valuable contributions have been made towards mobile and computer devices to monitor parameters [8,9,10,11,12,13], remote applications to monitor and control outputs [14,15,16,17,18,19], wireless networks and sensors [20], and smart implementations [21,22]. However, to integrate all the considered inputs and smart systems, a complex model is needed that enables users to intuitively make use of and monitor the available systems. On top of that, a simulated scenario for aquaponics will benefit from a deeper understanding of the correlations between parameters, caused by the symbiotic relationship between the biological systems, thus, leading to optimization and increased control over the system. In the authors opinion, developing a digital twin would support this research effort.

Digital twins (DTs) are commonly composed of three components: a physical entity, its virtual representation, and the communication channels between them [23]. DTs are typically adopted to improve the performance of physical entities by leveraging computational power and techniques and by using a virtual counterpart [24]. Within the Industry 4.0 ecosystem, DT offers an agile, smart, and cost-effective environment that enhances the systems’ productivity [25]. The origin of DTs is attributed to Michael Grieves and the work developed with John Vickers of NASA in 2003 [26]. The initial conception intended to provide the foundations for product life-cycle management for situations where the possibility of gathering data was scarce, manually performed, and/or limited by the available resources [26], with the potential risk of, eventually, putting the concept in standby. The interest in digital twins nowadays is mainly attributed to the advances in the technologies around the Industry 4.0 era, such as the Internet-of-Things (IoT), big data, real-time sensors, and big data management and processing techniques [27,28]. 

**Figure 1:**
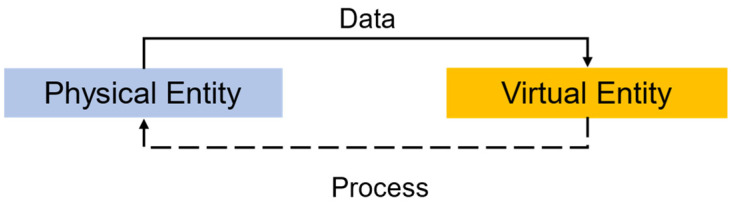
Digital twin schematic.

A representation of the basic principles behind a digital twin is displayed in Figure 1. Jones et al. conducted highly valuable work characterizing the digital twin concept through a literature review, facing the reality where the ideas around digital twinning were diverse and did not converge, mainly due to the disorganized rapid growth of the applications, limiting the nourishment of the area and the maturation of the concept [24]. As of today, a better and clearer understanding about the components of a DT and their interrelationships is considered, and the expected impact of future research will be higher and in a broad range of industrial applications.

This paper proposes to move forwards on the application of digital twins in aquaponics, following on from the current literature, and close the loop back to the physical system. A digital twinning process of the grow beds (hydroponics component) of an aquaponic system is developed. The main objective of the presented research is to create a virtual platform in which aquaponic practitioners can visualize the results obtained through sensors and smart systems in real time. For example, an interface is proposed to showcase the individual growth rate and fresh weight of crops estimated using an online tool based on image processing and deep learning segmentation [29]. Moreover, system parameters such as pH, electroconductivity, water temperature, relative humidity, environment temperature, and light intensity are recorded and linked to the performance metrics. From there, a database is created to serve as a base for the implementation of smart algorithms that relate performance metrics versus parameters to achieve optimal parameters. Finally, an exploratory analysis of the information is presented as an intuitive approach of the behavior of the system.

This paper is organized as follows: first, the related work is presented in Section 2; a generic framework of a digital twin is introduced in Section 3.1 based on the systematic review; next, Section 3.2 reports on the proposed framework for aquaponics; then, Section 4 encompasses the case study, experimental setup, and components used for this research; and, lastly, in Section 5, the results obtained as proof of concept are presented and discussed.

## 2. Related Work

The adoption of a digital twin is a valuable tool for the optimization of the process and labor reduction because it promotes the implementation of models for prediction, optimization, and the use of monitoring interfaces. It has been researched quite extensively over the years in the field of agriculture and precision farming. However, few contributions that researched to implement the concept of digital twins in agriculture as well as aquaponic systems can be found in the literature. The primary reasons for this are the complexity of system, the dependence on natural conditions (climate, soil, humidity) and the presence of living physical twins (plants and animals) and non-living physical twins (indoor farm buildings, grow beds, outdoor agricultural fields, agricultural machinery) [30]. A brief analysis of these DTs is presented in this section.

A model was constructed to simulate the behavior of the system under current monitored conditions (air temperature and humidity, light intensity, pH, electroconductivity, water level, water flow, and water temperature) [28]. The authors modeled some system characteristics such as the fish feed rate, the total dissolved solids in water, the fish weight gain, the water pH and nitrates, and plant growth. The author reported good estimations in most of the predicted models, except for the nitrates and plant growth. The main drawback of their proposed system lies in the lack of feedback from the real system in terms of plant growth and rate. This result is expected since the changing conditions of the environment and the ‘somehow’ changing-adapting behaviors of the living organisms alter their growth conditions continuously. In another study, a morphological framework is developed for a digital twin of a product-service-system to support potato harvesting [31]. To realize the foundations of DT in agriculture, Naoum et al. developed ‘AgROS’, an emulation tool based on the Robot Operating System, which could be used for assessing the efficiency of real-world robot systems in customized fields [32]. It allows farmers to select their actual field from a map layout, import the landscape of the field, add characteristics of the actual agricultural layout (e.g., trees, static objects), select an agricultural robot from a predefined list of commercial systems, import the selected UGV into the emulation environment, and test the robot’s performance in a quasi-real-world environment [32]. In another study, a digital twin was developed for smart vertical farming by building a cyber-physical system (CPS) so farmers can better understand the state of their farms regarding the use of resources and equipment [33]. The proposed system gathers data from the soil probe and displays its information in a dashboard which enables the further deployment of more soil probes and other monitoring and controlling devices to create a fully operating digital twin [33]. A digital twin model was developed by Jose et al. aiming at the joint creation of physical and digital layers of IoT-enabled structures for vertical farming to improve productivity, allow self-configuration to environmental changes, promote energy saving, ensure self-protection with continuous structural monitoring, and reach self-optimization learning from multiple data sources [34]. Payman et al. presented an ‘AgScan3D+’, an automated dynamic canopy monitoring system to generate a digital twin of every tree on a large orchard scale [35]. It consists of a spinning 3D LiDAR plus cameras that can be retrofitted to a farm vehicle and provides real-time on-farm decision support by monitoring the condition of every plant in 3D, such as their health, structure, stress, fruit quality, and more [35].

## 3. Research Methodology

The digital twin framework for aquaponics and the process developed in this paper is based on the available literature, which takes on consideration theory and review publications on digital twins [23,24,26], manufacturing processes [27,36,37], and farming implementations [28,29,30,31,32,33,34,35,38]. In the following subsections, the general concepts of digital twin implementation and its use to develop an aquaponics framework are explained.

The analysis in this paper is based on a comprehensive systematic review of the literature about digital twins. The objective of this research is to propose a framework of digital twinning that can be generally applied to aquaponic systems. For this purpose, quantitative methods are used; in this case, a systematic approach, experimentation, and validation using the circular approach of design research science. The systematic analysis is based on a qualitative analysis of selected journals and conferences that infers the framework proposed. The experimentation design and setup are done using the CropKing^®^ NFT Desktop System, Lodi, OH, USA. In this sense, the plant selected is Little Gem Romaine lettuce, which takes 14 days from seeding to harvest. Specific details about the experimentation procedure are defined in Section 3.2.

### 3.1. Digital Twinning Framework

**Figure 2:**
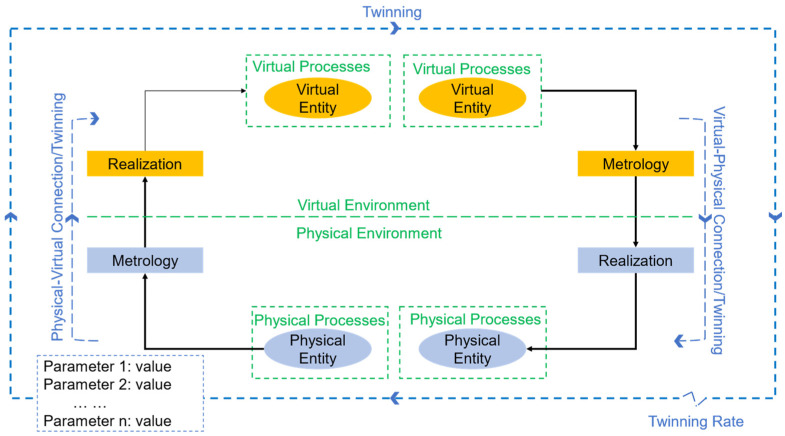
Digital twinning schematics, including main components and information flow (adapted from [24]).

The generic methodology for the construction of a digital twin framework and its application is based in Figure 2, which displays the schematics of the relationship between the digital twin components [24]. A twinning process is based on the virtual-to-physical and physical-to-virtual relationships in which identical scenarios are presented in both environments. The main concepts and processes needed for a digital twin are presented below.

The concept of physical and virtual entity refers to a specific real object, product, machine, or process and its virtual counterpart. Expanding the concept, the word environment is introduced for both cases (i.e., physical environment and virtual environment) that refers to the whole system and the internal relationships; not just limited to the level interactions (physical-to-virtual and virtual-to-physical). 

The parameters are another key component of a digital twin system, and it refers to the type of data and information of the processes that is passed between the physical and virtual environments. Fidelity and state are two attributes that describe the characteristics of the selected parameters. Fidelity describes the value and accuracy of each parameter, while state describes its current condition. 

The other two important components are the physical-to-virtual connection and the virtual-to-physical connection. In these connections lie the main differentiators of digital twins compared to other virtualization processes, i.e., simulation models. Physical-to-virtual connection is the communication and virtualization process: how the state of the physical entity or environment is transferred to the virtual parameters such as interfaces, graphs, databases, and so forth. There are two phases in this connection type, metrology and realization. Metrology refers to the method in which the parameters are captured (i.e., sensors) and sent to the virtual component, and realization refers to the virtualization approach used to update and execute accordingly regarding the parameter inputs. The continuous mode in how this connection is established is what makes this process an online interaction. The virtual-to-physical connection represents the flow of information that goes from the virtual side to the physical part. This information or analysis output enables the functionality in the physical system to adjust or change its processes, for example, to improve its performance. This connection is identical to its physical-to-virtual counterpart, with similar actions (metrology and realization) as aforementioned. In Jones’ opinion, this is the valuable paradigm of digital twins since it defines the bidirectional relationship between the virtual and the physical twins. However, the official definition of the digital twin by the CIRP encyclopedia of production engineering does not include this interaction as mandatory, which is also addressed by the same authors [39]. 

Physical and virtual processes are the specific activities performed on each of the levels, i.e., simulations, modelling, prediction, and optimization of virtual processes or manufacturing tasks, along with the control and design of physical processes. Finally, the twinning rate defines how the frequency at which the virtual and physical environments are synchronized, which leads to the consideration of digital twins as a real-time virtualization. 

In the next subsection, the generic framework just presented is implemented for aquaponic systems. Based on the concepts just introduced, these general components are defined in an aquaponic framework.

### 3.2. Aquaponics Digital Twin Framework

Aquaponics itself is a complex farming method due the also complex relationships between its components. Developing a digital twin framework addresses the need to have a better understanding of these relationships and how they can be improved. Following the framework aforementioned, the digital twin for aquaponic systems is discussed below and summarized in Table 1.

**Figure 3:**
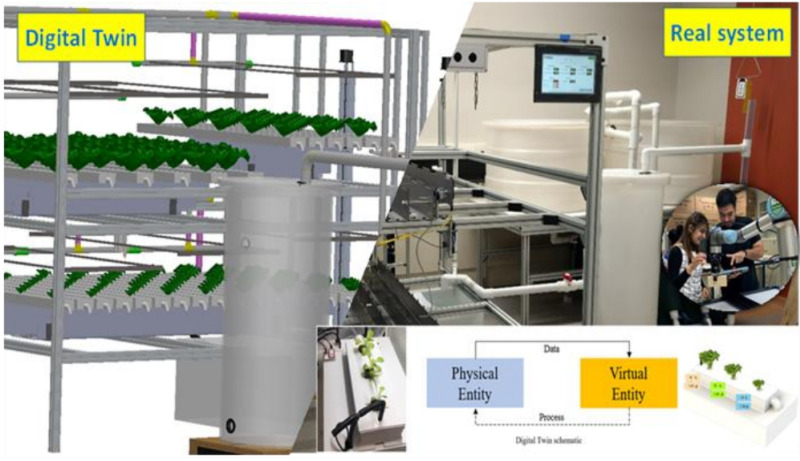
Digital twinning representation of the studied aquaponics system.

In an aquaponics system, the physical entity refers to the fish tanks, where the fish are farmed, and the grow beds. The virtual entity is a virtual representation of the physical entities. As such, 3D modeling software is often used to build these objects, machines, or processes in the virtual environment. The physical and virtual environments involve all the individual physical components and the virtual processes associated to them. For example, the physical environment includes the biofilters, pumps, feeders, aerators, humidifiers, sensors, etc., in addition to the physical entities previously mentioned. Then, the virtual environment includes the interface, buttons, notifications, graphs, tables, and so forth that establish a virtual dashboard or interface that represents the physical environment. A visualization of the physical and virtual environments used in this study can be found in Figure 3.

Parameters are an important component in any digital twinning process and extremely relevant to virtualize and simulate an aquaponic system [7]. Following the literature, several parameters are defined in aquaponics: pH, water and air temperatures, water level, dissolved oxygen, electroconductivity, total dissolved solids, salinity, total ammonia-nitrogen, nitrites, nitrates, flow, relative humidity, and light intensity, among others. In some cases, more complex dependent parameters obtained from the physical side can be included as parameters, for example, fish age, plant growth, or remaining available resources. Those parameters monitor the performance of the system in a more comprehensive fashion.

Physical-to-virtual and virtual-to-physical connections are mostly generic in a digital twinning process and depend more on the controllers and interfaces adopted rather than the aquaponics application itself. On the one hand, generic tools used in the physical-to-virtual connection can vary from industrial protocols and applications developed by hardware and software companies to open-source developments. For example, SQL, MySQL, or PHP are commonly used for the transfer and storage of the data acquired, to build procedures, and to control internal processes. Additionally, several IoT technologies are now available to build robust connections, e.g., wireless modules. On the other hand, the virtual-to-physical connection is mostly limited by the specific components chosen in the physical and virtual side, although on a few occasions, connections may be built using open-source communication protocols at the risk of having cybersecurity issues.

Further, the dependence of communication protocols and physical devices increases when considering the incompatibility of physical devices and certain software or communication protocols. For example, taking the commercial controllers from the ABB company (Allen Bradley PLC’s), the communication system will solely rely on RsLinx, which is the communication protocol from the company that allow their PLC to send and receive information and be controlled remotely. The initial selection of the physical system brand determines the protocols and software that will be used to generate the digital twin. Part of it can be avoided if the use of micro-controllers is implemented to control the physical environment. The development of the connection tools is easier since they are mostly open source and are available for the use with Raspberry Pi, Arduino, and other open-source controllers. Herein, the software or programming language that is used to build the interface is the main factor for the resources available, in which each case is different. 

Finally, aquaponics metrology is often constructed through a series of sensors for the parameters, cameras, mathematical, and smart models installed in the grow beds and fish tanks. Realization refers to the reactive effect of the metrology part and makes use of the controller used and the metrology applied, while the twinning rate defines the heartbeat of the system. To display sensor results, asynchronous updating of its values occurs every second; however, if more complex processes are developed, such as prediction tools or optimal parameters adjustments, elapsed times around 5 min is a good option. As aquaponic systems are slow dynamical processes, changes occur in the long term, enabling long synchronization of the digital twin, and easing the introduction of complex and tedious tasks.

## 4. Hydroponic Grow Bed Digital Twin

### 4.1. Case Study

**Figure 4:**
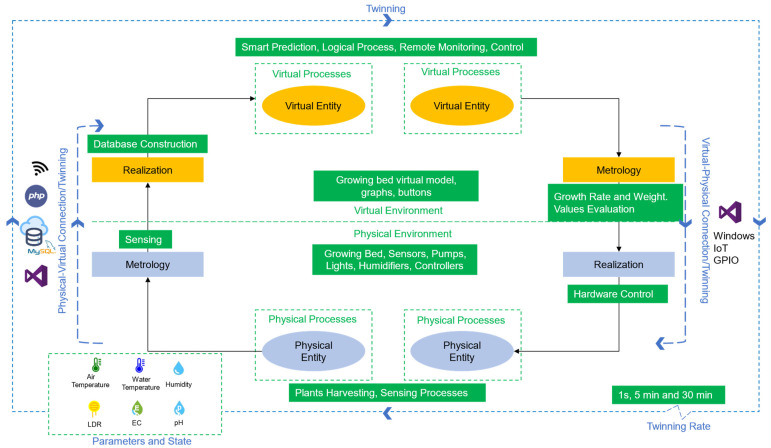
Proposed digital twin framework, after [24].

A case study is developed to prove the concept of the digital twin in aquaponics. The scope of the experimentation is limited to applying the concept in the hydroponics (grow beds) component of the aquaponics environment. For this case study, Figure 4 illustrates the specific aquaponic components using the framework provided in the previous section.

Physical entity refers to the grow beds, and virtual entity refers to the built model that mimics it. The physical environment are all the components listed in Section 3.2, and the virtual environment are the virtual entity, information displayed, and the graphical user interface (GUI). The parameters are the sensor measurements. The values of the components are considered the state of the parameters.

The physical–virtual connection is achieved using a wireless sensing module, php, MySQL, and the connection from the visual studio to the database in the network. Here, the metrology is the action of sensing the parameters, and the realization in the physical system corresponds to the database construction with the process of gathering and analyzing the raw data. The virtual–physical connection is executed using the IoT Core module and Visual Studio to control the outputs in the main controller. The metrology stage in this connection mode refers to the growth rate and fresh weight estimation using the prediction models and the logical process to evaluate the sensed values against the optimal levels. The realization, on the other hand, in this case, is the action to turn on or off the outputs depending on the conditions. Physical processes are specifically the growing of the plants and the processes involved (water flow, humidity, light), and the sensing of the parameters. Virtual processes correspond to the execution of the prediction process, the logical classification of the levels, and the remote monitoring and control of the system. Lastly, the twinning rate is determined by the one second for the display of the sensors values, the five-minute interval between the database records, and the thirty-minute time-lapse between predictions.

The project development will be explained following an explanatory process. First, the experimental setup will be described along the components installed and the experimental principles such as frequency and working logic. Secondly, the process to calculate the growing rate and fresh weight estimation will be shortly explained. Third, the process used to construct the database will be introduced. Fourth, the twinning interface and the tools to build it will be presented. Fifth, the feedback loop from the virtual to the physical environment will be introduced, and the performed actions will be explained in detail.

### 4.2. Experimental Setup

A hydroponic grow bed setup is designed and constructed for the experimentation part. The frame of the device was assembled using MDF (medium density fiber) board. This holds the NFT channel (grow bed) with a water pump and a heater, two cameras (one at the side and other one at the top of the grow bed), artificial growing lights, a camera ring light (to provide consistent light to the cameras), and a humidifier. As for the sensors introduced in the system, pH, electroconductivity, water temperature, air humidity, air temperature, and luminosity are used. For the main controller, a Raspberry Pi is selected, while an Arduino Nano is employed as the sensing unit. A list of all the experimental setup components is presented below:

- 1 CropKing^®^ NFT Desktop System.
- 1 Water Pump
- 1 Water Heater (25 degrees Celsius)
- 2 ELP 1080P Webcam (2.8–12 mm HD Lens)
- 1 Growth Light (T5 high output bulb, 24 W).
- 1 Camera Ring Light (56-LED Lamps)
- 1 Humidifier
- 1 Raspberry Pi controller 3B+
- 1 4-Channel Relay Module (110–220 V)
- 1 Arduino nano micro-USB Microcontroller
- 1 Liquid PH Value Detection Sensor
- 1 Analog Electrical Conductivity Sensor
- 1 DS18B20 Water Temperature Sensor
- 1 DHT22 Air Temperature and Humidity Sensor
- 1 LDR Sensor
- 1 ESP8266 Wireless Sensor
- 1 2-Channel Relay Module (5 V)
- 1 5 V Power Supply

The two cameras are connected to the Raspberry Pi and work with scheduled scripts that take pictures every 30 min from 6:00 am to 6:00 pm. The growing lights, the camera ring, and the humidifier are connected to the four-channel relay module which is triggered by outputs from the main controller. For the sensor modules, all of them are installed in an Arduino Nano with the corresponding configurations. The pH and electroconductivity sensors are connected to a two-channel relay module to avoid measuring problems. In addition, an ESP8266 Wi-Fi Sensor is installed in the Arduino, allowing the data to be transmitted wirelessly to the main controller (Raspberry Pi) and to the twining interface for real-time sensor values display.

### 4.3. Growth Rate and Fresh Weight Estimation

The estimation of the grow rate and fresh weight of the leafy crops is done through a smart implementation using a predictive model for the localization and multi-instance segmentation of the plants. The model was constructed using the MASK-RCNN framework proposed by He et al. [40]. The images acquired by the experimental setup are first conditioned to avoid radial and tangential distortion [41], and then are segmented using MASK-RCNN. From the prediction model, a script routine is created using Python to extract the parameters of interest which are listed in Table 2.

**Figure 5:**
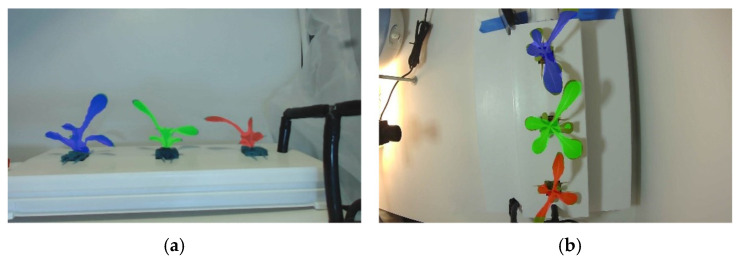
Example of segmented plant identification: (**a**) side image, (**b**) top image.

With these parameters, the overall height, width, and depth are calculated to derive the growing rate of the plants. A linear regression model is then created to describe the fresh weight of the plants, which is validated using experimental results. The fresh weight model is presented in Equation (1), and the values of linear regression coefficients are presented in Table 3. Sample images from the segmentation process are shown in Figure 5.

(1) $$Y=\beta_{0}+\beta_{1}{X\;}_{2}+\ldots +\beta_{n}{X\;}_{n}$$

The whole model validation and explanation can be found in a previous work; hence, it is recommended to consult it for further details [29].

### 4.4. Database

Building the database is a key step towards the construction of this digital twin model and future uses of the model in knowledge discovery in databases (KDD) techniques. Regarding the digital twinning process, the mechanisms to send and receive information, the fidelity and accuracy of the information, and the twining rate are defined in this step. As per the experimental setup, six sensors are used to monitor the current state of the system: pH, electroconductivity, water temperature, relative humidity, air temperature, and light intensity. To ensure the future deployment of the system at a larger scale, the sensing module is designed as an external device to the main controller; therefore, an Arduino Nano is implemented for this task. The limitation about connectivity of the Arduino Nano is overcome using an ESP8266 wireless sensor that allows the controller to send data wirelessly to the main controller, a Raspberry Pi.

**Figure 6:**
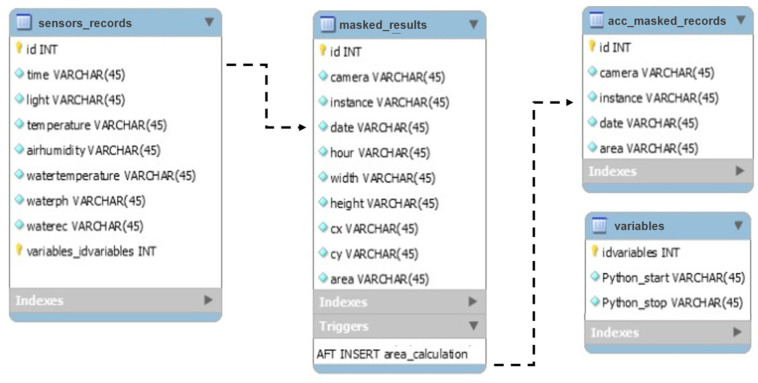
Model of the relational database implemented for the digital twin proposed for aquaponic systems.

The IoT sensing module processes the values from the different sensors and sends the information through a digital access point to the Raspberry Pi every five minutes. The main controller formats and inserts the values in a MySQL database at the local level using PHP. During this process, a host computer executes a parallel process using the growth rate and fresh weight models and sends the obtained results to the main database every 30 min, synchronously with the other records. Figure 6 shows the model of the relational database designed with the tables and relationships used.

**Figure 7:**
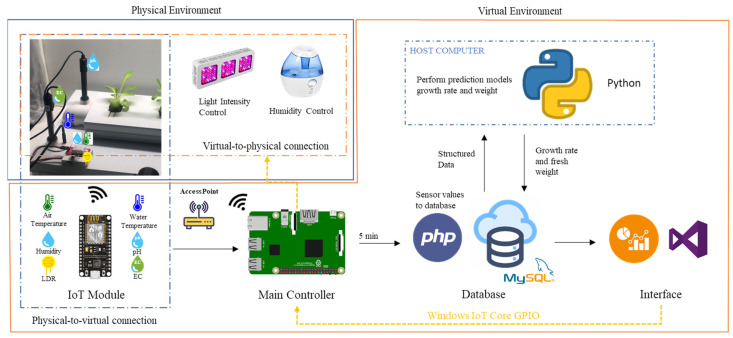
Schema of the digital twinning integration for the hydroponic grow beds of an aquaponic system.

In summary, a database that describes the current state of the system is available and can be accessed through an IP address from any point in the network. As such, this allows the communication between any devices in the network at any time. Further details are also described in a previous work [42]. Figure 7 displays the IoT sensing module interaction process. 

### 4.5. Twinning Interface

**Figure 8:**
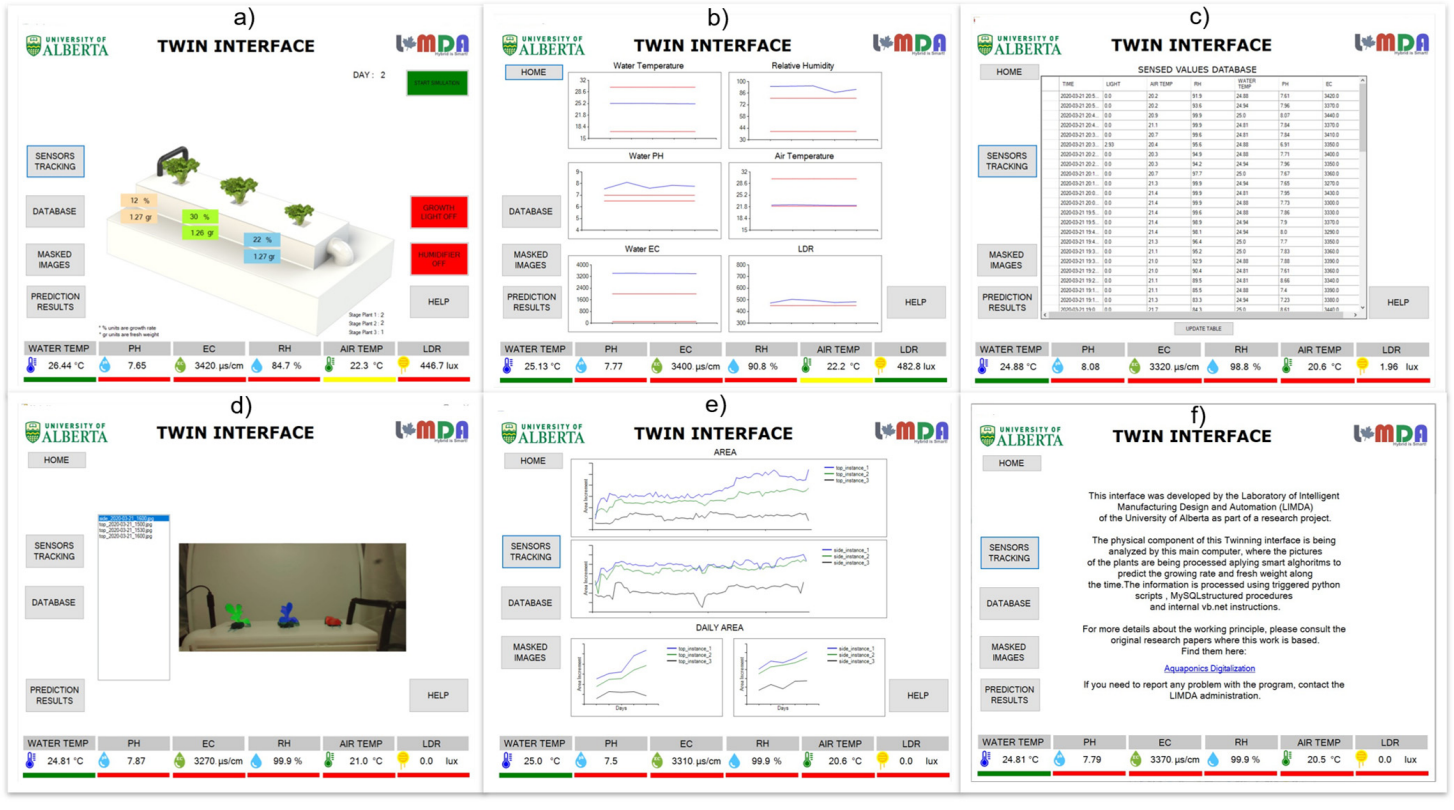
Digital twin interface: (**a**) ‘Home’ window; (**b**) ‘Sensors Tracking’ window; (**c**) ‘Database’ window; (**d**) ‘Imaging’ window; (**e**) ‘Predictions’ window; (**f**) ‘About’ window.

One of the main benefits of digital twins is its ability to close the gap between the user and the digital process [43]. This can be achieved by giving them a clear understanding about the state of the digital process and the internal activities performed. Introducing a visualization tool in the digital twin process removes the idea of a ‘digital black box’, giving them total control and supervision of the system at the physical and virtual levels. The twinning interface concept describes the virtual graphical display of the system. The graphical interface is designed using Visual Studio, and it is directly connected to the database to retrieve the information it needs for displaying. Additionally, a direct connection to the IoT sensing module is done via serial communication, allowing real-time sensed values to be retrieved every second. Six different windows are designed to offer the user organized and appropriate requested information, as shown in Figure 8, the different windows are ‘Home’, ‘Sensors Tracking’, ‘Database’, ‘Imaging’, ‘Predictions’ and ‘About’.

In the ‘Home’ window, a replica of the physical entity (NFT channel) is designed and acts as the main component of this interface. Interactive and resizable reproductions of the plants are modeled, and the current size of the crops in the physical side at real time are mimicked in the virtual environment. Information about the growth rate and weight of each of the plant is displayed. For the plant’s growth, a decision model is built to categorize the actual size of the plant and assign a stage from 1 to 12. This categorization is made by calculating the growth area along the 14 days from previous runs, averaging them and dividing them evenly throughout the 12 stages; thus, every stage corresponds to an area interval (i.e., stage 1: 0 cm^2^ to 1 cm^2^). Based on this level, the scalable digital model that represents the plant current growth status is displayed to the user. The second window is named ‘Sensors Tracking’, where tracking graphs for each sensor are displayed with the historical values of the sensors and is updated every 5 min synchronously with the ‘sensors-records’ table. Third, the ‘Database’ window is where the all the entries of the ‘sensors-records’ are displayed. Fourth, ‘Imaging’ gives the user the opportunity to visualize the segmentation output of the masked leafy vegetables, as seen in Figure 5, and to identify potential problems in the image processing results. As explained before, top and side pictures are taken of the plants every 30 min, with the purpose of extracting information about the state of the plants (see Table 1). Once a new set of pictures is available, a Python script is executed to run a prediction model to identify the plants’ location and extract the features of each of them using instance segmentation. At this point, the digital interface connects to a specific folder and retrieves the masked images to present them in the window ‘Masked images’ for the user. The features from the plants are saved into a MySQL table named ‘masked-results’. A structured procedure is designed into MySQL to be triggered by the insertion of a new value in this ‘masked-results’ table; therefore, it constantly calculates the average of the area, height, width, and depth features, grouping by view, instance, and date. This data is saved in a new table named ‘accumulative-masked-records’. Fifth, the ‘Predictions’ window is where the predictive models used show their results. This window displays, through graphs at the record level, the side and top area of each of the plants and the daily calculations automatically made by the MySQL procedure. The digital interface by itself calculates the growing rate based on the change in the area of the plant and the fresh weight of the plants using the values in named ‘accumulative-masked-records’, and the logistic linear model shown in Equation (1) and Table 2. Lastly, the ‘About’ window displays information about the working principle of the interface. 

**Figure 9:**
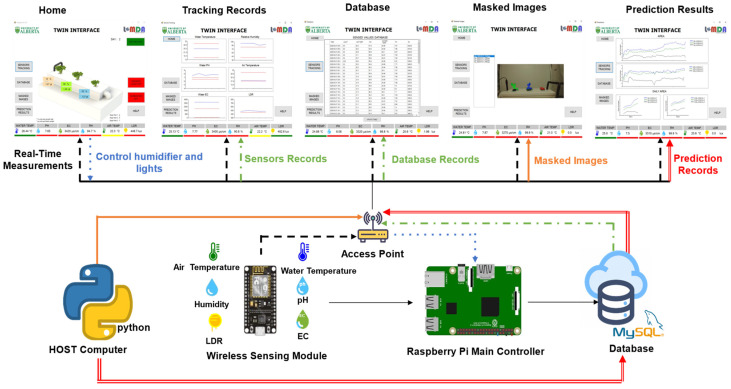
Communication between the interface, MySQL, and the physical component.

All the windows share a lower banner that displays the current values of the sensors that are retrieved directly from the sensing module every second. A logical procedure is then developed to showcase the status of the grow bed at that moment. On the other hand, this parameter’s values are saved every five minutes directly by the main controller (Raspberry Pi) into a MySQL table in the network called ‘sensors-records’. After this evaluation, a three-color code similar to ‘traffic lights’ displays the current status when compared to predetermined correct ranges. For each parameter, the color chosen is assigned as green if the value is between the acceptable levels, yellow if it ends near the maximum or minimum (15% from the limits), and red if the value is not conforming, displaying to the user not only the value, but also the meaning of the read measurements in terms of performance and adequate growth environment for the plants. A summary of those parameter ranges is presented in Table 4, from [7]. Figure 9 shows the communication between the interfaces.

### 4.6. Feedback Loop

The feedback loop refers to the virtual–physical connection and the way the virtual environment sends an action to be executed in the physical environment. This is possible using the IoT Core connection in Visual Studio. For this case study, simple orders for the feedback loop are designed to prove the concept: the main controller in the physical system is prepared with scripts to turn on and off the system growing lights, humidifiers, and ring lights for the cameras, as requested. 

**Figure 10:**
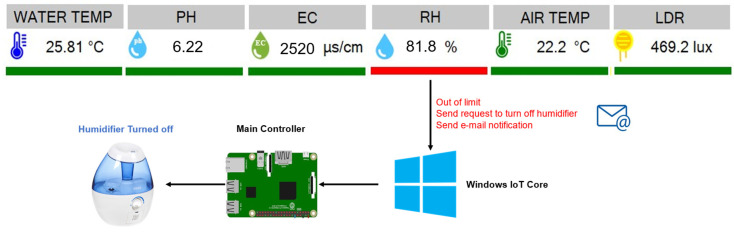
Working principle illustrating automation and control.

After the twinning model retrieves the sensor information and evaluates whether the levels in humidity or light intensity are in the correct range of values, it automatically sends a request to the Raspberry Pi to turn on, off, or adjust the specific device to reach adequate values. In addition, this action can be performed manually by a set of buttons in the interface. All of the automatic decisions and changes made by the digital twin are logged and notified to the operator of the system via e-mail. Figure 10 illustrates this working principle.

## 5. Results

### 5.1. Feedback Control

**Figure 11:**
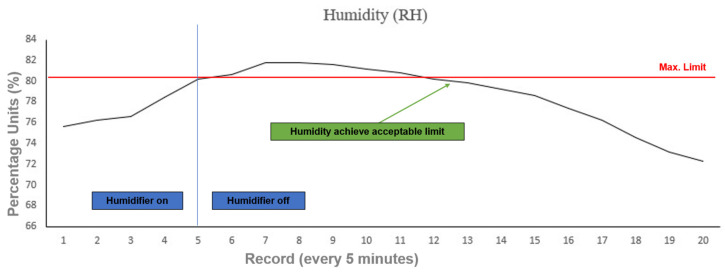
Relative humidity correction after sensor feedback and control response.

As a basic procedure to prove the working principle, and as a first step of the future work related to the autoregulation of an aquaponic system, the interface can autonomously turn on/off any actuators of the system, i.e., the humidifier or the growing lights, by sending an instruction to the Raspberry Pi through the IoT Core module in Visual Studio to activate or deactivate the relays (as shown in Figure 10). To validate this communication and test the virtual-to-physical connection, the order to turn off the humidifier is sent after detecting ‘humidity’ values out of the limits. Figure 11 shows relative humidity (RH) values during the reported experimental setup. As observed, once the current value of the humidity in the system reaches the maximum acceptable value of 80% (see Table 4), the humidifier is turned off. After a brief period of overpass, the system humidity starts to decrease progressively until it returns back to acceptable levels. The humidifier will remain off until the lower limit for acceptable humidity (50%) is reached, at which point it will be turned on again.

The objective of all the control systems in any aquaponic setup should aim to correct the parameter values in the minimum amount of time in order to reduce its negative effects on the plants’ growth. Depending on the parameter selected, differences in time response can be critical to maintain a healthy environment [44,45]. The presented example is, in no fashion, an accurate representation of an optimized solution to control the humidity in the aquaponic environment. However, it presents a clear interface that permits the testing and optimization of such controls, either by directly accessing the physical systems or by creating a simulated environment based on the entries stored in the database.

### 5.2. Discussion

**Figure 12:**
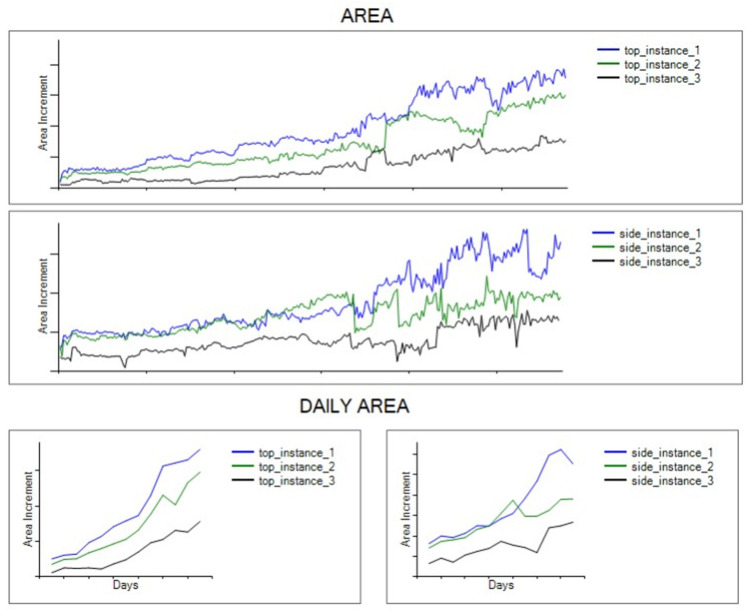
Visualization of the area trends in plants, based on overall system growth (**top**) or individual instances (**bottom**).

Through the continuous analysis and monitoring of the data, it is possible to get a better understanding of the growing behavior of the plants. The presented system proves to be reliable at identifying trends and linking parameters of the environment to two design performance metrics, which is the initial step towards complex implementations. The research community and the aquaponics/hydroponics practitioners will highly benefit from this contribution. Figure 12 displays the growing behavior of the three different plants throughout the whole growth process and on a daily basis. With this implementation, the user is constantly aware of the status of the sensors and, consequently, the plants, and can turn on/off system actuators at will, such as lights and humidifiers. Furthermore, live data can be analyzed almost instantaneously with the use of KDD algorithms that, for example, allow the system to estimate the growth rate of the plants and predict their weight. As such, a complete understanding of the process can be presented with not only the status of the parameters, but also the incidence of them in the outputs (performance metrics). 

From the results obtained during the experimentation, it is noted that plant #1 had an overall growth rate of 15%, with a side growth rate of 14.7% and a top growth rate of 15.6%. The average growth rate in plant #2 was 14.46%, the side growth rate was 10.25% and the top growth rate was 18.67%. For plant #3, the average growth rate was 23%, with a side growth rate of 22.73% and a top growth rate of 23.16%. As observed, plants growing under the same conditions theoretically do grow at different rates. One of the limitations of the presented study is that some design and physical aspects of the aquaponics system, such as the air flow in the system or the distance between plants, are not included. This limits the potential modeling of the growth rate of the plants based on the data obtained using the proposed system. Extension of the proposed system to include these factors, among others, will be pursued by the authors in the near future.

Further analysis can be done by comparing, not only the daily growth rates, but the daytime growth rates versus the nightly growth rates. In this study, it was found that plants grow faster during the morning/afternoon, with an average growth rate of 28%, while growth was reduced to an average of 16% during the evening/night. Similarly, analyzing other records from the sensing values during the experimentation, it is interesting to find that the pH values of the water are slightly higher during the morning with an average of 0.15 difference, with average values of 6.9 and 7.05, respectively. The opposite effect can be found regarding the electroconductivity, where higher values are consistently found during the nights, with average of 2500 µS/cm and 2550 µS/cm, respectively. Another finding, which is more intuitive, is about the relative humidity behavior: higher values are found at night, with values around 15–20% higher in comparison with the morning hours. As such, based on the empirical evidence for the plants studied, different resources’ consumption and control strategies might be needed for different hours of the day, while keeping the objective of maximizing the growth of the plant at the end of the day and throughout the whole growth process. 

Some limitations about this framework and system can be addressed. A generic digital twin framework is adapted to the aquaponics technology; however, the scope is limited to the resources available: the hydroponics beds. Including the fish tank and corresponding systems, namely biofilters, feeding systems, etc., of the aquaponics system in future developments is necessary to finalize the complete digital twinning of the process. Monitoring the weight of the fish (density in the tanks), assessing their health, and including the inherent associated parameters, such as ammonia transformation and dissolved oxygen, will definitely open more possibilities regarding the control and optimization of the process. Finally, the digital twin aquaponics framework can be validated as a whole in future developments.

## 6. Conclusions

Aquaponics is becoming a popular method as a sustainable solution for indoor food production. Under the background of ‘Industrial 4.0′, digital twinning technology has been widely used as a tool to realize the interaction and interconnection between physical and virtual spaces. This paper describes the framework and implementation of the digital twin technology for the hydroponic component of an aquaponic system. By integrating IoT technology, databases, control strategies, artificial intelligence, and visualization tools, the virtual model of the physical system can be created, updated to reflect status changes in real time, and represents a comprehensive reference for aquaponic users.

The presented study showcases the use of the digital twin platform to acquire data in real time, make use of data-driven algorithms to determine growth rate and fresh weight, and make informed decisions towards a healthy aquaponic environment. Ultimately, it presents a platform towards optimizing crop yield in aquaponic systems. The benefit of this paper for research and practitioners is the provision of an integrated detailed framework for digital twinning, which is to be enriched to determine optimal functionality of aquaponic systems and provide clear performance metrics that support commercialization of the aquaponics technology.

## Figures and Tables

**Table 1:** List of generic components in the aquaponics digital twin framework.

Component	Framework Definition	Aquaponics Framework
Physical entity	Specific real object, product, machine, or process physically present	Fish tanks and/or grow beds
Virtual entity	Replica of the existing physical entity into the virtual world	3D models or virtual representations of the physical entities
Physical environment	All the physical entities and the relationships between them	Physical entities plus pumps, lights, humidifiers, water treatments, sensors, etc.
Virtual environment	Virtual entities and the tools to display them such as graphs, buttons, interfaces, models, etc.	Interfaces, graphs, tables, buttons, notifications, etc.
Parameters	Parameters that define the behaviour of the physical system and help the virtual environment to perform the mimicking	Including, but not limited to, pH, electroconductivity, RH, ammonia, nitrites, nitrates, light intensity, etc.
States	State of the parameters, can be defined in terms of values, levels, stage, etc.; fidelity and state are inherent adjectives of the state	Values of the related parameters for the physical entities
Physical-to-virtual connection (PVc)	How the data is transferred from the physical to the virtual environment	IoT technologies such as wireless modules, SQL, programming languages, among others
Virtual-to-physical connection (VPc)	How the data is transferred from the virtual to the physical environment	Type of physical and virtual controller, i.e., RsLinx for the ABB controller
Metrology	Measuring the state of the parameters in either of the physical or virtual environments	Sensors, cameras, etc., for PVc; evaluation tools and mathematical models for VPc
Realization	The actions that the correspondent environment take to adjust/change based on the metrology input	Databases, dashboards, notifications, etc., in the PVc; hardware control, changes in levels, etc., in the VPc
Physical processes	Processes executed in the physical environment	Seedling, harvesting, feeding, water treatments, etc.
Virtual processes	Processes executed in the virtual environment	Smart prediction models, data tracking and recording, levels adjustments, etc.
Twinning rate	Rate at which the interaction between environments is performed	Commonly ‘real-time’ for critical processes; in non-critical processes, defined elapsed time may range from 5 min to 30 min

**Table 2:** List of features extracted.

Plant Features	
Side view area	$A_{side}$
Height	$H_{side}$
Width	$W_{side}$
Centroid side	$C_{xys}$
Top view area	$A_{top}$
Centroid top	$C_{xyt}$
Depth	$D_{top}$

**Table 3:** List of linear regression coefficients.

Plant Features	
β_1_ Height	−0.000859
β_2_ Depth	+0.001044
β_3_ Width	+0.005135
β_4_ Side Area	−0.000007
β_5_ Top Area	+0.000042
β_0_ Intercept	+0.246012

**Table 4:** Optimal parameters for aquaponics experimentation.

Parameters	Aquaponic
pH	6.5–7.0
Electroconductivity	100–2000 µSiemens/cm
Water Temperature	17–30 °C
Relative Humidity	50–80%
Air Temperature	22–30 °C
Light Intensity	>450 lux

## Data Availability

Not applicable.
